# Delusion-like thinking is associated with lower individual alpha peak frequency

**DOI:** 10.1038/s41537-025-00626-w

**Published:** 2025-05-19

**Authors:** Luca Tarasi, Domenico Romanazzi, Anna Pasini, Vincenzo Romei

**Affiliations:** 1 Centro studi e ricerche in Neuroscienze Cognitive, Dipartimento di Psicologia “Renzo Canestrari”, Università di Bologna, Cesena, Italy; 2 Universidad Antonio de Nebrija, Madrid, Spain

**Keywords:** Neural circuits, Biomarkers

## Abstract

Schizophrenia and schizotypy are understood to lie along a continuum of neurophysiological and cognitive features, yet the specific neural markers bridging clinical and subclinical manifestations have remained underexplored. In our study (*N* = 318), we found that reduced Individual Alpha Frequency (IAF)—previously established as a neural marker in schizophrenia—features magical thinking trait in schizotypy. This finding broadens the relevance of IAF to subclinical populations linking it to delusion-like thinking in schizotypy and suggests its potential as a transdiagnostic indicator across the schizophrenia spectrum.

Schizotypy is a multidimensional construct that encompasses subclinical traits such as diminished social connections, altered perceptions, and cognitive eccentricities^1^. While these traits remain below the threshold for clinical diagnosis, they closely parallel core features of schizophrenia^2^. Recognizing this overlap, psychiatry has increasingly adopted a dimensional framework placing schizotypal traits along a continuum of Schizophrenia-Spectrum Disorders (SSD^3^). This continuum perspective is supported by converging evidence from genetic and behavioral studies, revealing shared biological underpinnings between schizotypy and schizophrenia^4–6^.

Despite the growing body of research investigating parallels between schizotypy and schizophrenia, few studies have explored their neurophysiological similarities in terms of brain oscillatory activity. One promising marker in this domain is the IAF, defined as the peak frequency at which alpha oscillations (7–13 Hz) reach their maximum amplitude. Alpha rhythms play a critical role in cognitive functions such as attention, memory, and sensory integration^7^, and the IAF has emerged as an indicator of cognitive and perceptual efficiency. For instance, higher IAF is linked to enhanced perceptual accuracy and cognitive performance^8,9^. Crucially, a lower IAF has been consistently observed in schizophrenia^10^ and associated with cognitive deficits, including delayed perceptual processing^11^, impaired visual processing^12^, and generalized cognitive dysfunction^13^.

Here, we tested the hypothesis that IAF could act as a shared neural hallmark of SSD, potentially linking schizotypy and schizophrenia. To explore this hypothesis, we conducted a large-scale study using the largest EEG dataset in the field (*N* = 318) to determine whether schizotypal traits are associated with lower IAF. All participants signed a written informed consent before taking part in the study, which was conducted in accordance with the Declaration of Helsinki and approved by the Bioethics Committee of the University of Bologna (Protocol Code 201723, approved on August 26, 2021). All participants were drawn from the general population, did not report neurocognitive or psychiatric disorders and were not under neuro/psychiatric medication. Participants completed the Schizotypal Personality Questionnaire (SPQ^14^), a widely used self-report instrument designed to assess schizotypal traits across multiple dimensions—including positive, negative, and disorganized domains. Concurrently, resting-state EEG was recorded for 2 min with participants keeping their eyes closed. For each participant, IAF was extracted using the algorithm proposed by Corcoran et al.^15^. We then investigated whether there was a relationship between the IAF and SPQ and its subscale scores. Our findings indicate a trait-specific pattern of alpha oscillations associated with systematically lower frequency linked to the positive schizotypy trait magical thinking. Notably, regression analyses revealed that individuals with higher levels of magical thinking exhibited a lower alpha frequency, particularly within fronto-parietal regions (Fig. 1, *r* = −0.14, *p* = 0.03), while no difference emerged when considering individual alpha power (*r* = 0.04, *p* > 0.44). This pattern was further confirmed by a median-split analysis, which uncovered a marked, widespread decrease in IAF across cortical regions among individuals with elevated magical thinking scores (Fig. 2, High Mag = 10.18 ± 0.07 Hz, Low Mag = 10.30 ± 0.08 Hz, *p* = 0.02). IAF differences of this magnitude have previously been associated with variability in perceptual performance, suggesting that such shifts are functionally relevant and may critically influence the fidelity of external sensory sampling^9,16^. Intriguingly, prior research has documented comparable IAF reductions (~0.1 Hz) in first-degree relatives of individuals with psychosis compared to healthy controls^11^, raising the possibility that these subtle frequency shifts may index latent vulnerability markers.

**Fig. 1:**
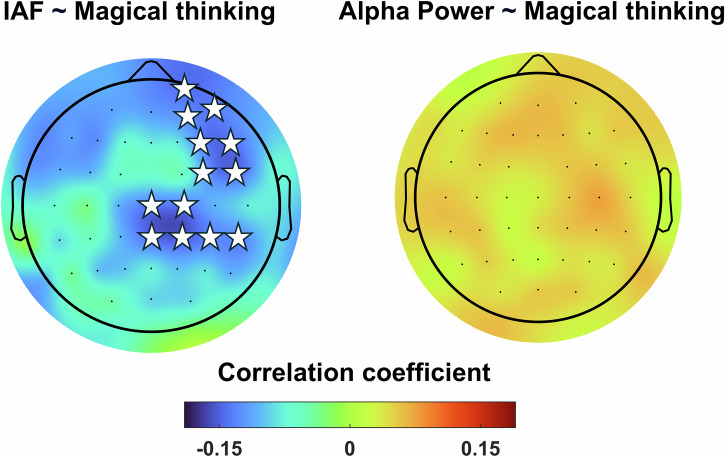
Higher levels of magical thinking are associated with lower IAF values in fronto-parietal regions, while no correlations emerged with Alpha power. Stars represent the cluster’s significant electrodes emerged from the regression analysis.

**Fig. 2:**
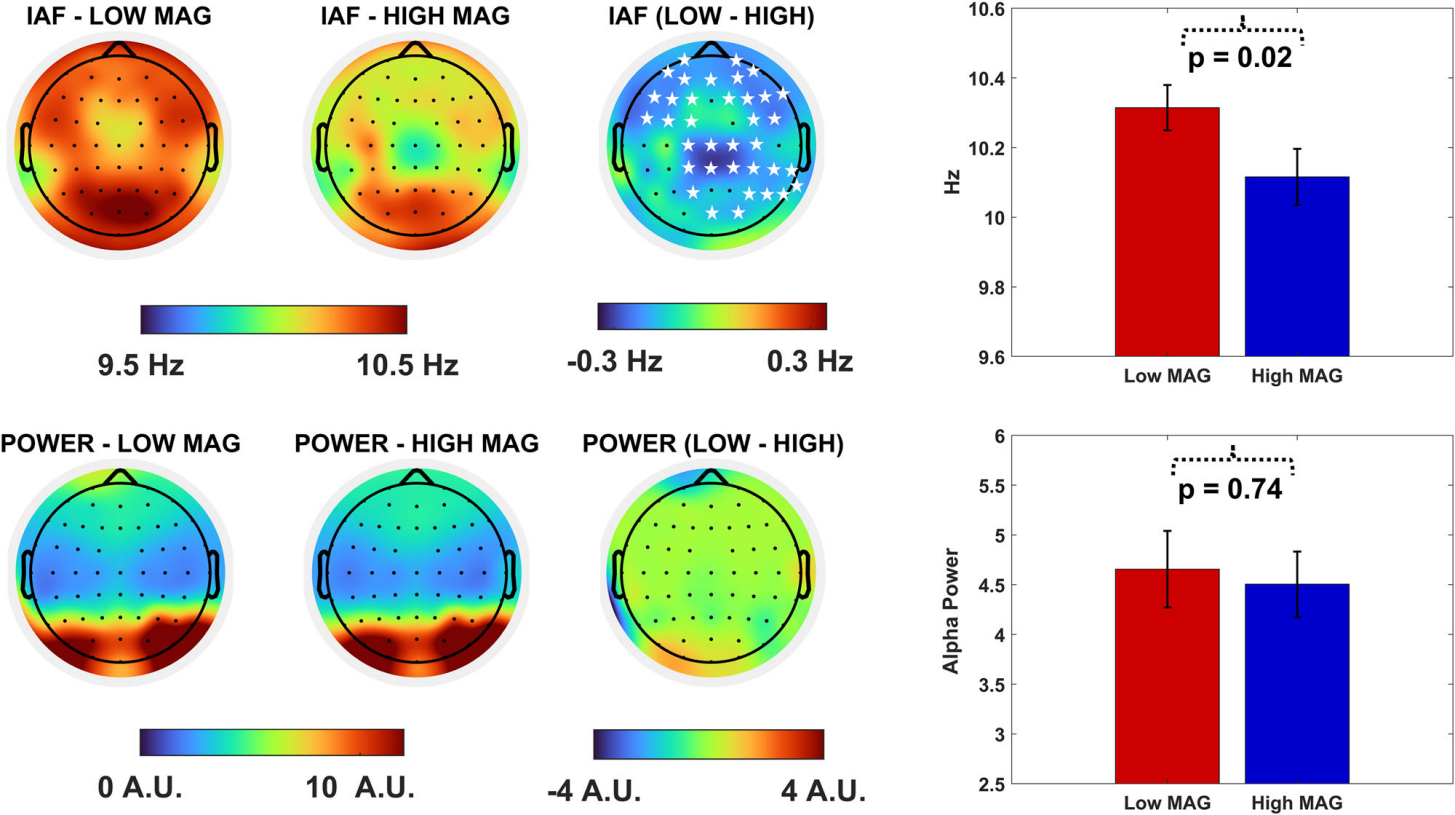
A significant widespread difference in IAF is observed between individuals with high and low levels of magical thinking, while no such difference emerges for Alpha power. Stars represent the cluster’s significant electrodes emerged from the differences in IAF between individuals with high and low levels of magical thinking.

Characterized by superstitions, paranormal beliefs, and anomalous perceptions, magical thinking is a hallmark of positive schizotypy and is closely linked to delusion-like thought patterns, reflecting a tendency to perceive connections and significance where none may exist. It constitutes a key feature of attenuated psychosis syndrome—a condition described in the DSM-5 as a potential precursor to psychosis^1^.

The specificity of the observed effect can be interpreted within the predictive coding framework. In the SSD population, a lower IAF has been associated with inefficient, impoverished sensory encoding^13^. Moreover, recent evidence indicates that people with high schizotypy exhibit aberrant functional connectivity and disrupted brain network organization^17^. This suggests that the combination of diminished sensory sampling—stemming from a lower IAF—and a compromised capacity for coherent information integration creates conditions in which the external world is perceived as fragmented, thereby fostering maladaptive, delusional interpretations^18^. Future research could test this interpretation through perceptual decision-making tasks involving predictive processing. Additionally, future ontogenetic studies could explore the causal relationship between IAF deficits and connectivity abnormalities in schizotypy, potentially positioning IAF impairment as a critical factor leading to the emergence of disorganized neural patterns.

Notably, while lower alpha frequency was linked to magical thinking, no association was observed with traits related to negative or disorganized symptoms. This finding is consistent with evidence that the positive and negative components of schizotypy are often dissociated, exhibiting strikingly divergent patterns at neural, behavioral, and psychometric levels^19,20^.

Importantly, the reduction in alpha frequency was predominantly observed in fronto-parietal regions. This finding aligns with previous evidence pointing to anatomical and functional alterations within the fronto-parietal network in individuals with schizotypal traits^21–23^. Furthermore, it is consistent with EEG studies on SSD, which have reported a global reduction in IAF, with the strongest effects typically emerging in fronto-parietal areas^13^.

The implications of these findings are significant. Alpha oscillations are integral to a wide range of cognitive functions^9^, and a lower IAF has been consistently linked to cognitive impairments in schizophrenia^13^. By demonstrating similar alterations in individuals with positive schizotypy, our findings suggest that alpha dynamics could help identify individuals at potential risk for SSD. This, in turn, may support early diagnostic efforts by providing a neurophysiological marker associated with vulnerability before clinical symptoms fully manifest.

Moreover, the trait-specific association with neural indices underscores the value of focusing on symptom dimensions rather than solely on categorical diagnoses. This approach aligns with contemporary frameworks such as RDoC, which prioritize transdiagnostic neural markers over traditional diagnostic boundaries^24^.

## Supplementary information

SUPPLEMENTARY MATERIAL

## Data Availability

Data available to corresponding authors upon reasonable request.
