# Supplementary material for: Delusion-like thinking is associated with lower individual alpha peak frequency
Source: Schizophrenia (Heidelb). 2025 May 19;11(1):76. doi: 10.1038/s41537-025-00626-w (PMC12089434; doi:10.1038/s41537-025-00626-w)
Supplement: Supplementary file 1 — SUPPLEMENTARY MATERIAL [file 41537_2025_626_MOESM1_ESM.docx]

**Participants**

The sample consisted of 318 participants (198 female; age range: 18–35 years). All participants signed a written informed consent before taking part in the study, which was conducted in accordance with the Declaration of Helsinki and approved by the Bioethics Committee of the University of Bologna (Protocol Code 201723, approved on August 26, 2021). All participants reported no neurocognitive or psychiatric disorders. Focusing on a non-clinical, non-medicated sample allowed us to investigate interindividual variability in schizotypal traits along a subclinical continuum, without potential confounds related to medication use, clinical impairment, or illness chronicity.

**Schizotypal Personality Questionnaire**

The Schizotypal Personality Questionnaire (SPQ) (Raine, 1991) is a self-report formed by 74-item divided in 9 subscales: 1) Ideas of reference (excluding delusions of reference): interpreting benign remarks as having hidden or threatening meanings; 2) Odd beliefs or magical thinking: holding unconventional beliefs that influence behavior, such as, superstition, clairvoyance, telepathy or "others can feel my feelings”; 3) Excessive social anxiety: experiencing profound discomfort in social situations involving unfamiliar individuals; 4) Unusual perceptual experiences: experiencing illusions, sensing a presence, or feeling that things, oneself or the world are unreal (derealization and depersonalization); 5) Inappropriate or constricted affect: exhibiting behavior that seems overly silly or distant, and rarely mirroring social gestures or facial expressions, such as smiling or nodding in return; 6) No close friends or confidants (or only one) with the exception of first-degree relatives: having few or no friends and often preferring isolation; 7) Odd or eccentric behavior or appearance: being unkempt, displaying unusual mannerisms, self-talk, or wearing inappropriate or peculiar clothing; 8) Odd speech (without loosening of associations or incoherence): using digressive, vague, or overly abstract language, with unconventional or idiosyncratic word choices; 9) Suspiciousness or paranoid ideation: believing that others may be plotting against them or harboring ill intentions. The original scoring methods were implemented by assigning to each response a binary code (0/1). Given that each subscale assesses distinct and specific dimensions (e.g., cognitive, perceptual, social, interpersonal, and behavioural dysfunction; DSM-5, American Psychiatric Association, 2013), the analyses were conducted separately for each.

**Recording, preprocessing, IAF extraction**

EEG was recorded at rest for 2 minutes, while participants kept their eyes closed. A 64-electrode setup was mounted according to the International 10–10 system. EEG was measured with respect to FCz electrodes, with all electrode impedances maintained below 10 kΩ. EEG signals were acquired at a rate of 1000 Hz and processed offline using custom MATLAB scripts (Version R2022b) in combination with the EEGLAB toolbox (Delorme & Makeig, 2004). The EEG recording was filtered offline in the 0.5- to 70-Hz band. Visual inspection was performed to identify noisy channels which were subsequently spherically interpolated, and recording segments corrupted by artifacts were eliminated. The recording was then referenced to the average of all electrodes. Independent Component Analysis (ICA) was applied to identify and remove artifacts that were clearly distinguishable from brain-origin EEG signals. To calculate the peak alpha frequency (IAF) we applied the algorithm proposed by Corcoran, Alday, Schlesewsky, and Bornkessel-Schlesewsky (2018). While others approach have methodological problem such time consuming and vulnerable to inconsistencies arising from subjective evaluation or risk of producing spurious or biased estimates under certain plausible spectral conditions, this method combines a common technique for automated detection of local maxima (i.e., searching for first-derivative zero crossings) with a widely recognized approach for resolving spectral peaks (i.e., Savitzky-Golay filtering) to derive an estimate of IAF. The average alpha peak across electrodes was centered at 10.12 ± 0.05 Hz.

**Brain-to-behaviour: Correlation analysis**

To examine the association between the extracted IAF values and SPQ, as well as each SPQ subscale, we first conducted a correlation analysis. Specifically, for each electrode, we computed the Spearman correlation coefficient between IAF values and each schizotypy subscale. Spearman’s method was selected because it does not assume a linear relationship and is robust to outliers. This analysis provided a set of correlation coefficients and corresponding p-values for each electrode and subscale. Next, we applied a cluster-based permutation approach to address the issue of spatial multiple comparisons. To do so, we identified clusters of neighboring electrodes that exhibited significant correlations (p < 0.05). Clusters were defined as groups of contiguous electrodes showing significant effects, with spatial adjacency determined by the electrode layout. To assess the statistical significance of the observed clusters, we conducted a permutation test. Specifically, we shuffled the relationship between IAF and the schizotypy subscales (n = 1000 permutations) while maintaining the original data structure. For each permutation, we recalculated the correlations and identified clusters of significant electrodes. This generated a null distribution of cluster-level p-values. Finally, we determined the significance of the observed cluster size by comparing it to the null distribution. A cluster was considered significant if its size exceeded the 95th percentile of the null distribution (p < 0.05). The significant cluster that emerged from the regression analysis, which revealed an association between higher levels of magical thinking and lower alpha frequency, includes: CP6, CP2, Cz, FC6, F4, Fp2, CPz, CP4, C2, FC4, F6, AF8, AF4​. Moreover, we address the issue of multiple comparisons across the nine SPQ subscales. To this end, the cluster-level p-values computed for each subscale were adjusted using MATLAB’s *mafdr* function, which implements Storey’s FDR procedure ^5^. Crucially, the association between Magical Thinking and Individual Alpha Frequency remained statistically significant after this correction (p-value FDR = 0.047).

To rule out a potential age-related confound, we repeated the correlational analysis using partial Spearman correlations, controlling for participants' age. The results confirmed that the significant negative association between IAF and magical thinking remained unchanged in both topography and extent, supporting the robustness of the effect independently of age.

To verify that magical thinking was exclusively associated with IAF and not with other components of alpha oscillations, we also analyzed the relative alpha power. Transforming the power spectral density into relative power allowed for more meaningful comparisons across individuals by accounting for individual differences in overall power. Specifically, we calculated relative power by dividing the summed power spectral density in the alpha range by the total power spectral density across all frequency bins within the range of 1 to 40 Hz for the selected electrode. Importantly, no significant association was found between magical thinking and relative alpha power, as no clusters emerged from the analysis.

**Brain-to-behaviour: Median split analysis**

Given that the correlational analysis conducted earlier revealed a significant association between magical thinking and IAF, we performed an additional analysis to further support these findings. Specifically, we compared IAF differences across electrodes for individuals with extreme scores on the magical thinking subscale. To achieve this, we performed a median split analysis, dividing participants into two groups: those with low magical thinking scores (IAF low MAG = 10.30 ± 0.08 Hz) and those with high magical thinking scores (IAF high MAG = 10.18 ± 0.07 Hz). Independent samples Mann-Whitney U tests were conducted at each electrode to assess whether the high magical thinking group exhibited lower IAF values compared to the low magical thinking group. To address the multiple comparison issue, we further validated the significance of this effect through a cluster-based permutation analysis, as described earlier. Specifically, we computed the independent samples Mann-Whitney U tests to investigate IAF differences between the above- and below-median groups for each electrode separately. We then assessed the number of neighboring electrodes that showed a significant relationship (p < 0.05, one-tailed). To determine the statistical significance of the cluster, we conducted a permutation test (n = 1000), in which we shuffled the relationship between IAF and the two groups and calculated the number of neighboring electrodes showing a significant effect for each permutation. Finally, we compared the observed cluster size (i.e., the number of contiguous significant electrodes) to the null distribution derived from the permutation analysis to determine if the observed cluster was statistically significant. The significant cluster that emerged from the median-split analysis, which shows a significant widespread difference in IAF between individuals with high and low levels of magical thinking, includes: Fp1, F3, F7, FC5, CP1, P4, P8, TP10, CP6, CP2, Cz, C4, FC6, F4, F8, Fp2, AF7, AF3, AFz, F5, FT7, FC3, C1, P1, POz, PO4, P6, CPz, CP4, TP8, C2, FC4, F6, AF8, AF4, F2.

Finally, to rule out age as a potential confounding factor in this group-level comparison, we tested whether the two groups differed in age. An independent sample t-test revealed no significant age difference between the low and high magical thinking groups (t_316_ = -0.53, p = 0.60), indicating that the observed effects are unlikely to be driven by age-related differences.

**Reference**

1. Raine, A. VOL. 17, NO. 4, 1991 The SPQ: A Scale for the Assessment of Schizotypal Personality Based on DSM-III-R Criteria.
2. American Psychiatric Association. *Diagnostic and Statistical Manual of Mental Disorders*. (American Psychiatric Association, 2013). doi:10.1176/appi.books.9780890425596.
3. Delorme, A. & Makeig, S. EEGLAB: an open source toolbox for analysis of single-trial EEG dynamics including independent component analysis. *Journal of Neuroscience Methods* **134**, 9–21 (2004).
4. Corcoran, A. W., Alday, P. M., Schlesewsky, M. & Bornkessel‐Schlesewsky, I. Toward a reliable, automated method of individual alpha frequency (IAF) quantification. *Psychophysiology* **55**, e13064 (2018).
5. Storey, J. D. A Direct Approach to False Discovery Rates. *Journal of the Royal Statistical Society Series B: Statistical Methodology* **64**, 479–498 (2002).
